# Opportunistic screening for diabetes mellitus and hypertension in primary care settings in Karnataka, India: a few steps forward but still some way to go

**DOI:** 10.12688/f1000research.22825.1

**Published:** 2020-05-06

**Authors:** Pracheth Raghuveer, Tanu Anand, Jaya Prasad Tripathy, Abhay Subhashrao Nirgude, Mahendra M. Reddy, Subhashree Nandy, Habeena Shaira, Poonam Ramesh Naik

**Affiliations:** 1 Department of Community Medicine, Yenepoya Medical College , Yenepoya (Deemed to be University), Mangaluru, Karnataka, 575018, India; 2 Indian Council of Medical Research (ICMR), Department of Health Research, Ministry of Health and Family Welfare,, New Delhi, Delhi, 110029, India; 3 Department of Community Medicine, All India Institute of Medical Sciences (AIIMS), Nagpur, Maharashtra, 441108, India; 4 Department of Community Medicine, Sri Devraj Urs Medical College, Sri Devraj Urs Academy of Higher Education and Research (SDUAHER), Kolar, Karnataka, 563103, India

**Keywords:** early detection, lifestyle diseases, opportunistic screening, operational research, SORT IT

## Abstract

**Background: **Opportunistic screening for individuals aged ≥30 years at all levels of healthcare for early detection of diabetes mellitus (DM) and hypertension (HTN) is an integral strategy under the national program to control non-communicable diseases. There has been no systematic assessment of the screening process in primary care settings since its launch. The objective was to determine the number and proportion eligible for screening, number screened, diagnosed and treated for DM and HTN among persons aged ≥30 years in two selected primary health centres (PHCs) in Dakshina Kannada district, Karnataka, India during March-May 2019 and to explore the enablers and barriers in the implementation of screening from the perspective of the health care providers (HCPs) and beneficiaries .

**Methods: **This was a sequential explanatory mixed-methods study with a quantitative (cohort design) and a descriptive qualitative component (in-depth interviews and focus group discussions) with HCPs and persons seeking care. Those that were not known DM/HTN and not screened for DM/HTN in one year were used to estimate persons eligible for screening.

**Results: **Of 2697 persons, 512 (19%) were eligible for DM screening, 401 (78%) were screened; 88/401 (22%) were diagnosed and 67/88 (76%) were initiated on treatment. Of 2697, 337 (13%) were eligible for HTN screening, 327 (97%) were screened, 55 (17%) were diagnosed with HTN; of those diagnosed, 44/55 (80%) were initiated on treatment.  The documentation changes helped in identifying the eligible population. Patient willingness to undergo screening and recognition of relevance of screening were screening enablers.  Overworked staff, logistical and documentation issues, inadequate training were the barriers.

**Conclusion: **Nearly 19% were eligible for DM screening and 13% were eligible for HTN screening. The yield of screening was high. We noted several enablers and barriers. The barriers require urgent attention to reduce the gaps in delivery and uptake of services.

## Introduction

Non-communicable diseases (NCDs) kill 41 million people each year (71% of global deaths), disproportionately more (>75%) in the low-middle-income countries ^1^. NCDs also account for a large share (75%) of deaths among those aged 30–69 years ^2^. They are a threat to the Agenda for Sustainable Development 2030, which targets reduction of premature deaths from NCDs by one-third ^3^.

India mirrors the global picture, with NCDs claiming 63% of all deaths in 2016 alone ^2^. India has nearly 72 million persons with diabetes mellitus (DM), which accounts for 49% of global burden and 207 million people with hypertension (HTN) ^4– 7^.

Early identification and prompt management through an emboldened health system is the key to reduce premature mortality and morbidity due to NCDs ^8^. To achieve this, India launched the National Programme for Prevention and Control of Cancer, DM, Cardiovascular Diseases and Stroke (NPCDCS) in 2010 ^9^. In Karnataka state, NPCDCS was introduced in a phased manner in various districts, starting from 2010–11 ^10^. In 2018, the programme was rolled out in Dakshina Kannada (DK) district, a coastal district in Karnataka. Opportunistic screening for persons aged ≥30 years at all public health facilities from sub-centres (SCs), primary health centres (PHCs) and above is an integral strategy for early detection of DM and HTN under the NPCDCS ^9^.

There has been no systematic assessment of the screening process in programmatic settings, with previous studies conducted in project settings ^11– 13^. Furthermore, their focus was on the yield of screening. It is operationally important to know how many of the eligible population, could be screened, which to our knowledge has not been previously addressed ^11, 13^.

Therefore, we conducted the present study among persons aged ≥30 years seeking health care from the outpatient department (OPD) of the selected PHCs in DK district of Karnataka from March to May, 2019 to determine i) the number and proportion eligible for screening of DM and HTN and ii) among those eligible, how many were screened, diagnosed and managed for the disease. Further, we qualitatively explored the enablers and barriers in the implementation of opportunistic screening from the perspective of the health care providers (HCPs) and persons availing the services.

## Methods

### Study design

This was a sequential explanatory mixed-methods study with a quantitative component (cohort study) and a descriptive qualitative component ^14^.

### Setting

***General setting***. Karnataka is the eighth largest state of India and is inhabited by 61.1 million with a literacy rate of 75.4% and is divided into 30 administrative districts ^15^.

DK, a coastal district of Karnataka, has a population of ~2.1 million and a literacy rate of 85.3%. It is divided into nine administrative divisions called Talukas ^16^. The prevalence of DM and HTN in DK are 16% and 17% respectively, higher than the national figures ^17, 18^.

***Specific setting***. Mangaluru is a predominantly urban Taluka of DK district with a population of ~1 million and a literacy rate of 91% ^19^. It has 22 PHCs and 12 urban primary health centres (UPHCs) which deliver primary health care to the population. We selected one UPHC located in Bunder, which caters to a population of 6,749 and one PHC located in Amblamogaru, a rural area with a population of 16,920. Yenepoya Medical College, where the Principal Investigator (PI) works, supports these centres by posting medical interns, as per a Memorandum of Understanding with the District Health and Family Welfare Office, DK.

*Opportunistic screening process for DM and HTN at the PHC level*

The PHCs run a general OPD where the basic demographics, diagnosis and treatment details are recorded in the OPD register. Under the NPCDCS, opportunistic screening is being conducted by the staff nurse under the supervision of the Medical Officer (MO) and details are recorded in a separate register (NCD register).The laboratory technician plays a supporting role in opportunistic screening for DM by carrying out tests like random blood glucose (using a glucometer) and fasting blood sugar (FBS), and maintains records of the tests conducted. An additional NCD related activity being carried out in these PHCs include population-based screening (PBS). PBS is carried out by accredited social health activists (ASHAs) through home visits in their service areas and by auxiliary nurse midwives (ANMs) at the SC level.

Monthly reports of all NCD-related activities at the PHC level are collated in a reporting format which captures details like cumulative number of persons screened, diagnosed, treated and on follow-up care for DM, HTN and other NCDs. This report is submitted to the district NCD cell, which is responsible for effective implementation and supervision at the district level. The NCD cell is managed by the District Programme Coordinator of NPCDCS, who works under the overall supervision of the District Surveillance Officer (DSO).

### Study population

For the quantitative phase all persons aged ≥30 years availing primary health care from the two selected PHCs from March to May 2019 were included. We excluded persons aged <30 years who sought primary health care from the two selected PHCs.

For the qualitative phase, HCPs working in the two selected PHCs, who were involved in screening for DM and HTN like staff nurses (n=4), laboratory technicians (n=2) and MO of the PHCs (n=3) were included. HCPs who were not involved in the screening process for DM and HTN at the two PHCs were excluded. The District Programme Coordinator, NPCDCS (n=1) was also interviewed. Persons who underwent DM and HN screening in the two PHCs from March to May 2019 (n=37) also constituted the study population.

### Data variables, sources of data and data collection

***Phase 1: Quantitative data collection***

*Setting up of a system for better documentation of opportunistic screening for DM and HTN at the selected PHCs*

Experiences from the field show that the existing recording system to document opportunistic screening carried has certain limitations, particularly with respect to determining the population eligible for screening among persons aged ≥30 years. It is not well documented whether a person has undergone screening previously or is already diagnosed as DM or HTN.

Thus, we set up a system to improve the existing documentation for opportunistic screening of DM and HTN.

After obtaining necessary permissions and building initial rapport with the HCPs, we conducted a stakeholder meeting at the PHCs. The limitations of the current recording system were discussed and additional variables were included in both the OPD and NCD registers. The variables include: a) whether the person has DM/HTN, b) whether screened for DM/HTN in the last one year. If the response to both a) and b) were “no”, the person was considered to be eligible for screening. The was done to estimate the number of persons eligible for screening and to assess the feasibility of this strategy in such settings. Further, we made amendments in the NCD register to collect certain essential information.

The staff nurses and laboratory technicians were trained to enter the required information in dichotomous responses (Yes/No). This enabled us to assess the eligibility for screening.

*Data collection*

The screening process was implemented by the HCPs from March to May 2019 at the two PHCs. To mitigate bias, none of the members of the study team were in contact with the HCPs of the two PHCs during the above-mentioned period of implementation. Thereafter, we collected details from the OPD and the NCD registers for the duration, March to May 2019 in a structured data collection proform a (available as *Extended data* ^20^) which had two parts. Data for the first part were extracted from the OPD register and data for the second part data came from the NCD register. The first part collected demographic details and eligibility criteria for screening. The second part collected information on whether persons were screened, diagnosed or managed for DM and HTN. Epidemiological diagnosis for DM, HTN and eligibility for screening are given in Table 1.

**Table 1.** Epidemiological diagnosis used in the study.

Variable	Epidemiological diagnosis
**Diabetes mellitus (DM)**	DM screening was being carried out using glucometers and a random blood sugar reading of >140 mg/dl was confirmed by fasting blood sugar. A fasting venous blood sugar level of ≧≥126 mg/dl was considered as DM. Fasting was defined as no caloric intake for at least 8 hours ^21^
**Hypertension (HTN)**	Blood pressure was measured using sphygmomanometers in the right arm, sitting position. A blood pressure of ≥140/90 mm of Hg with at least two measurements, five minutes apart was labelled as HTN ^22^.
**Eligible for screening**	Persons aged >30 years who are not diagnosed to have DM/HTN previously or not screened within the last one year in the PHC or community

***Phase 2: Qualitative data collection***. Systematic qualitative enquiry was carried out through key informant interviews (KIIs) among HCPs and focus group discussions (FGDs) among persons aged ≥30 years, who underwent screening for DM and HTN.

The PI has a master’s degree in Community Medicine/Public Health and is trained in qualitative research methods. The investigators were not a part of the programme implementation team.

The PI conducted the KIIs among HCPs at their workplace in Kannada (vernacular language), or English as applicable, until information saturation was attained. Participants were explained the purpose and their expected role prior to the interview about. Interview guides consisting of broad open-ended questions and probes were prepared for different cadres. Each KII lasted for around 30 minutes. Interview and FGD guides are available as *Extended data* ^33^.

The PI also conducted FGDs among persons aged ≥30 years, who underwent screening at the PHC. A total of 6–8 participants were included in each FGD. FGDs. Each FGD lasted for about 45 minutes and were held in Kannada language separately for men and women.

Only the participants, the PI and the note-maker were present during the KIIs and FGDs. Audio recording and verbatim notes were taken. In case the participants did not consent for audio recording prior to the discussion, notes were taken. After the KII/FGD was over, the summary was read back to the participants to ensure validation. A total of two repeat interviews were conducted among a staff nurse and laboratory technician working in one of the two PHCs. A repeat FGD was conducted among men aged ≥30 years who underwent screening for DM and HTN in one of the PHCs.

### Statistical and data analysis

***Quantitative data***. Quantitative data were double-entered and validated using Epi Data version 3.1 for entry. The data was analysed using Epi Data version 2.2.2.183 (Epi Data Association, Odense, Denmark) and STATA (v12.1) software.

**Figure 1.**
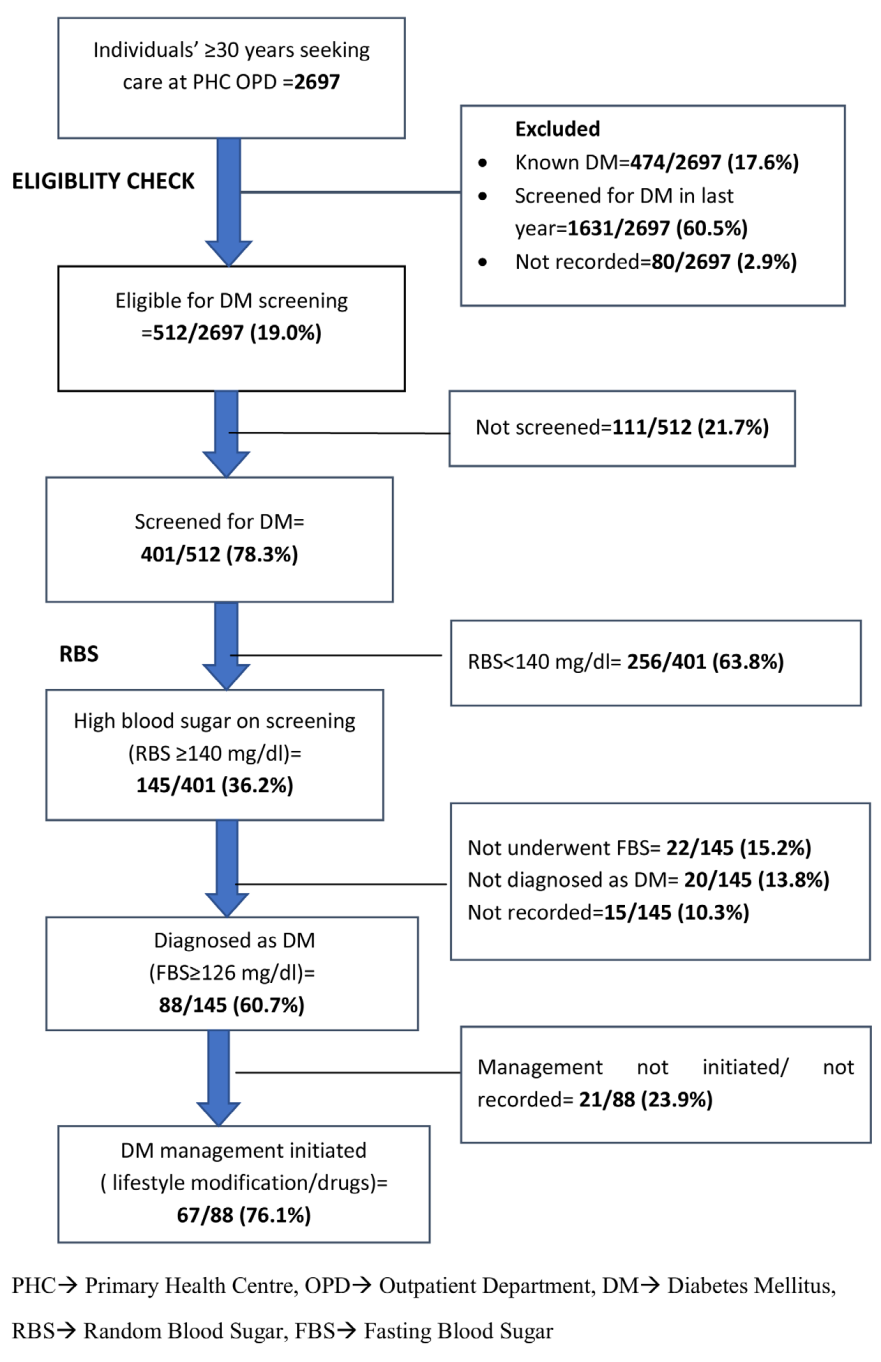
Flow diagram depicting the number eligible, screened, diagnosed and management for Diabetes Mellitus (DM) among persons aged ≥30 years seeking health care in the two selected primary health centres (PHCs) from March to May 2019.

**Figure 2.**
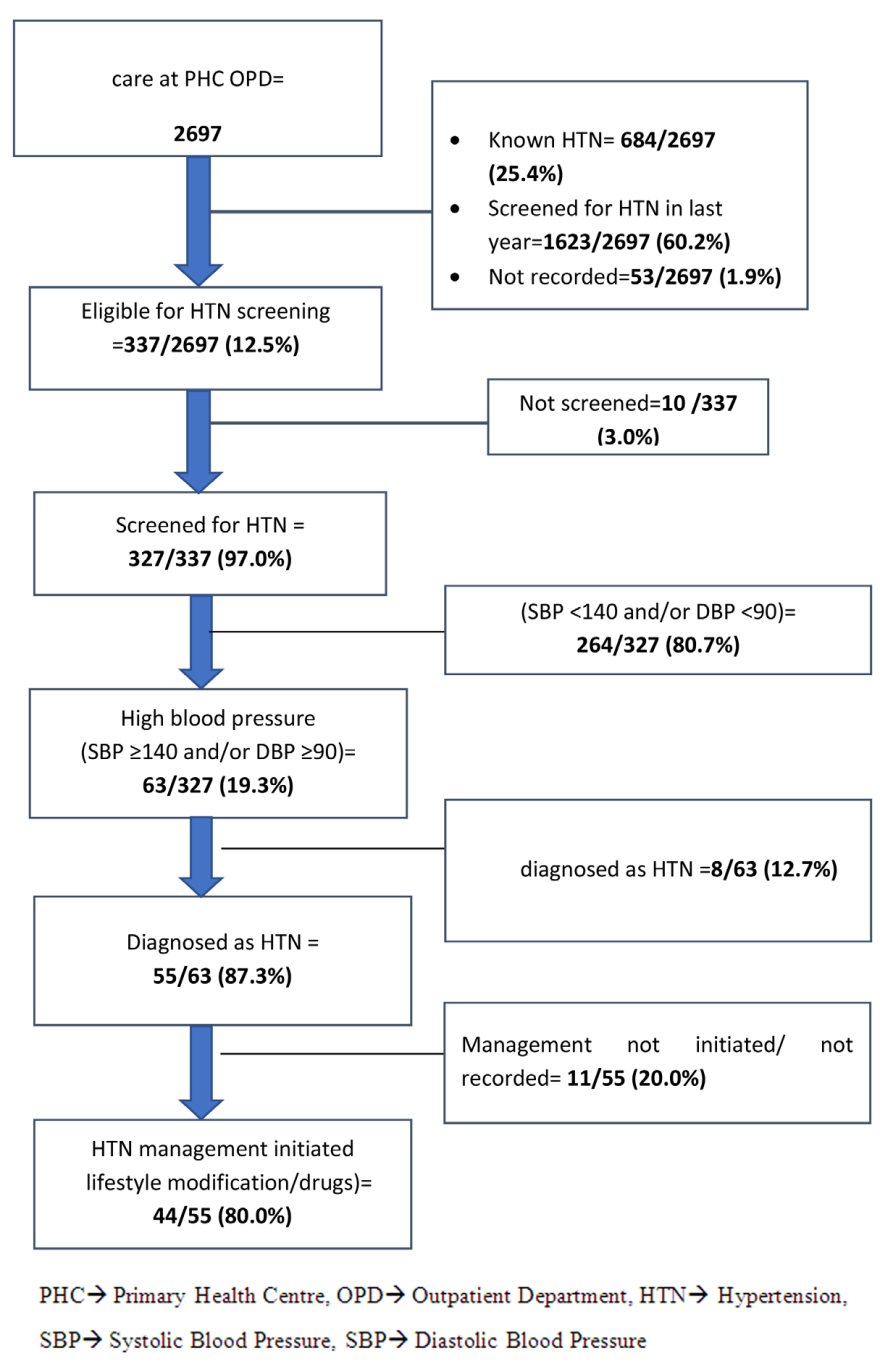
Flow diagram depicting the number eligible, screened, diagnosed and management for hypertension (HTN) among persons aged ≥30 years seeking health care in the two selected primary health centres (PHCs) from March to May 2019.

Continuous data were summarized using mean and standard deviation (SD). Categorical data were summarized as proportions. Key indicators like proportion of eligible population screened, diagnosed and managed for DM and HTN are presented in a flow diagram ( Figure 1 and Figure 2). To assess the factors associated with ‘not screened for DM and HTN’, we used Poisson regression. Adjusted relative risks (aRRs) with 95% confidence intervals (95% CIs) were calculated to eliminate the confounders. A p value of <0.05 was considered as the criterion of statistical significance.

**Figure 3.**
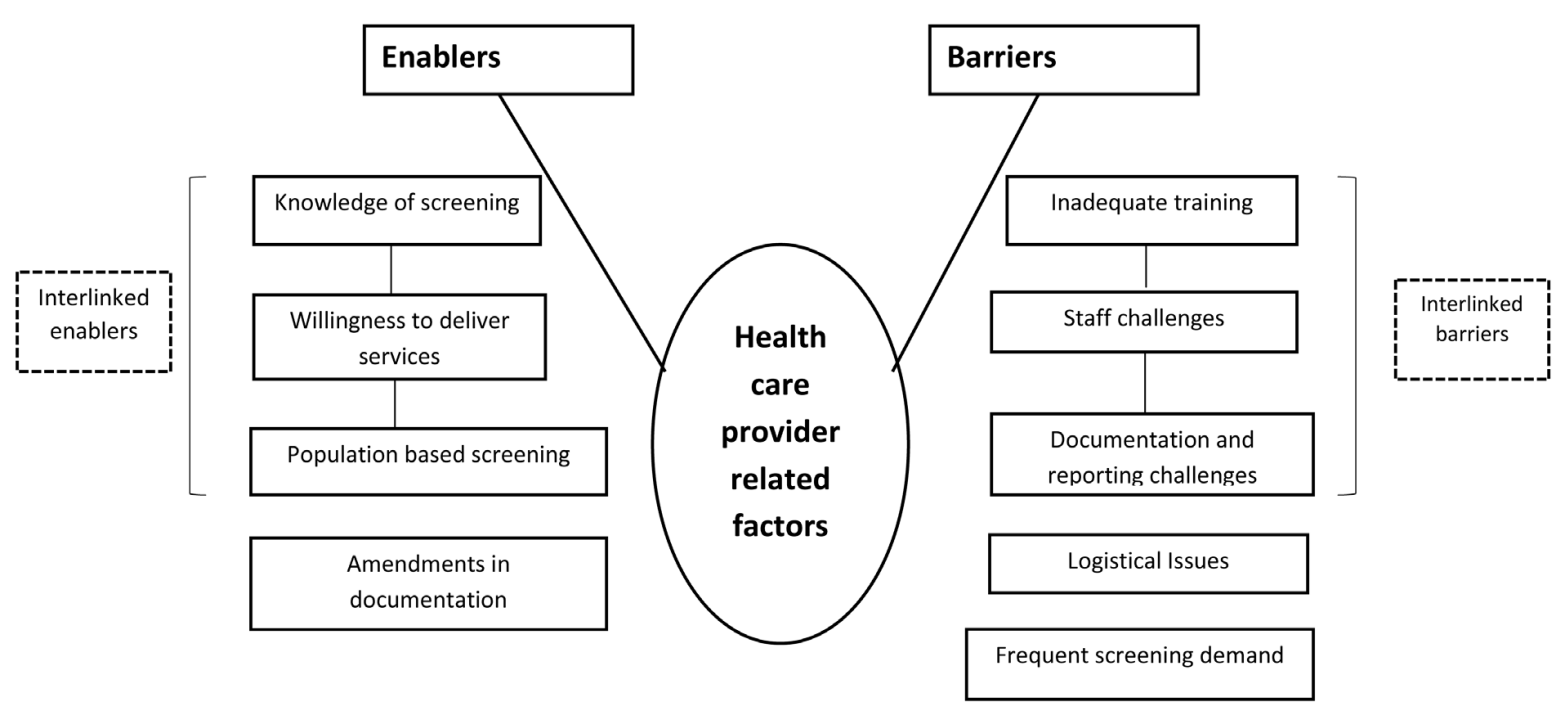
Non-hierarchical thematic map showing enablers and barriers in the implementation of opportunistic screening for diabetes melitus and/or hypertension among persons aged ≥30 years seeking health care at the outpatient department in the two selected primary healthcare centres from March to May 2019, as perceived by health care providers.

**Figure 4.**
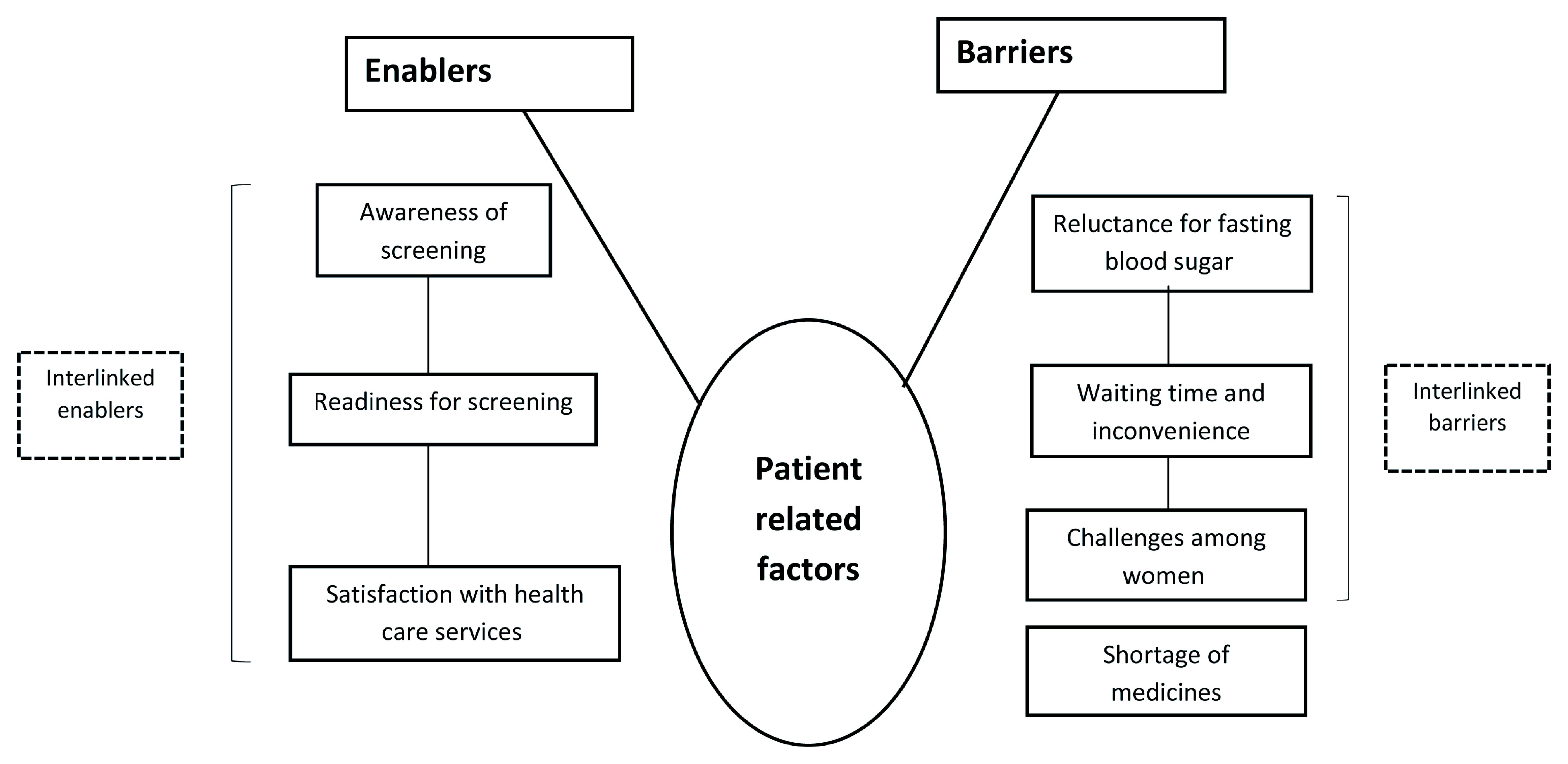
Non-hierarchical thematic map showing enablers and barriers in the implementation of opportunistic screening for diabetes mellitus and/or hypertension, as perceived by persons aged ≥30 years who underwent screening in two primary healthcare centres from March to May, 2019.

***Qualitative data***. The audio-recorded interviews and FGDs were transcribed by the PI (PR) in Kannada within 48 hours. Thematic analysis by manual coding was carried out by three researchers (PR, ASN and SN) independently to generate various categories or themes under the broad topics: HCP-related and patient-related enablers and barriers. Any discrepancy in coding was resolved through discussion and referral back to the audio files if necessary. If the discrepancy was still not resolved, a third investigator (PRN) reviewed the transcripts and codes. The transcripts and analysis were reviewed by other investigators (TA, JT) to reduce subjectivity in analysis and increase interpretive credibility. The codes were then organised into categories and common themes and presented in flow diagrams ( Figure 3 and Figure 4). A mix of inductive and deductive coding was done. Verbatim quotes are also presented (translated into English) within double quotations ^23, 24^. To ensure confidentiality, we have deliberately not mentioned the designation of HCPs in the quotes. The findings have been reported by using ‘Consolidated Criteria for Reporting Qualitative Research’ (COREQ) guidelines ^25^.

### Ethics and consent

Ethics approval was received from Yenepoya Ethics Committee-1,Yenepoya (Deemed to be University), Mangaluru (2019/085)and the Ethics Advisory Group of the International Union Against Tuberculosis and Lung Disease, Paris, France (126/18). Written informed consent was obtained from the study participants interviewed. Permission to carry out the study was obtained from the District Health and Family Welfare Officer, DK district.

## Results

### Participant backgrounds

Of the total 4120 persons seeking health care, 2697 fulfilled the eligibility for the study and were included in the analysis.

Of the 2697, 812 (30.2%), were aged 30–39 years with a mean age of 47.7 years (SD:12.3 years). More than half of the respondents were males (1525, 56.5%); nearly half were from UPHC (1350, 50.0%) ( Table 2).

**Table 2.** Socio-demographic characteristics among persons aged ≥30 years seeking health care in the two selected primary health centres (PHCs) from March to May 2019 (N=2697).

Variable	Total (N=2697)		Urban PHC (n=1350)		Rural PHC (n=1347)	
	N	%	n	%	n	%
**Age group (years)**						
30–39	812	30.2	381	28.2	431	32.0
40–49	760	28.2	408	30.2	352	26.1
50–59	549	20.4	299	22.1	250	18.6
≥60	565	20.9	255	18.9	310	23.0
Not recorded	11	0.3	7	0.5	4	0.3
**Gender**						
Women	1167	41.4	478	35.4	689	51.2
Men	1525	56.5	869	64.4	656	48.7
Not recorded	5	2.04	3	0.2	2	0.1

Of the 2697, 1631 (60.5%) were reported to have been screened for DM in the last year. A total of 512 (19%) were eligible for DM screening, among which 401 (78.3%) were screened for DM of whom, 88 (21.9%) were diagnosed as DM. Of the 88 diagnosed as DM, 67 (76.1%) were initiated on treatment ( Figure 1).

Of the 2697, majority (1623, 60.2%) had already been screened for HTN in the last year. A total of 337 (12.5%) were eligible for HTN screening. Of the 337, 327 (97%) were screened for HTN, of whom, 55 (16.8%) were diagnosed with HTN. Of the 55 diagnosed with HTN, 44 (80%) were initiated on treatment ( Figure 2).

In the adjusted analysis, female gender (aRR: 1.3, 95% CI: 1.0-1.8, p-value 0.04) was independently associated with ‘not being screened for DM’ ( Table 3). Male gender (RR: 2.4, 95% CI: 0.5-10.9, p-value 0.3) was not significantly associated with ‘not being screened for HTN’ ( Table 4). De-identified participant information is available as *Underlying data* ^26^.

**Table 3.** Association of socio-demographic characteristics with not being screened for diabetes mellitus (DM) among persons aged ≥30 years seeking care at the outpatient department in the two selected primary healthcare centres from March to May, 2019 (N=512).

Variable	Total	Not screened for DM		RR	95% CI	p-value	aRR	95% CI	p-value
		N	%						
**Age group (years)**									
30–39	239	62	(25.9)	1			1		
40–49	155	29	(18.7)	0.7	(0.5-1.1)	0.09	0.8	(0.5-1.2)	0.2
50–59	66	09	(16.1)	0.5	(0.3-1.0)	0.03	0.6	(0.4-1.3)	0.2
60 and above	52	11	(17.7)	0.8	(0.5-1.4)	0.5	0.8	(0.4-1.3)	0.4
**Gender**									
Men	320	61	(19.1)	1			1		
Women	192	50	(26.0)	1.4	(1.0-1.9)	0.06	1.3	(1.0-1.8)	0.04
**Residence**									
Urban	303	61	(20.1)	0.8	(0.6-1.2)	0.3	0.9	(0.6-1.2)	0.3
Rural	209	50	(23.5)	**1**			1		

aRR, adjusted relative risk; CI, confidence interval.

**Table 4.** Association of socio-demographic characteristics with not being screened for hypertension (HTN) among eligible population seeking care at the outpatient department in the two selected primary healthcare centres from March to May, 2019 (N=337).

Variable	Total	Not screened for HTN		RR	95% CI	p-value	aRR	95% CI	p-value
		N	%*						
**Age group (years)**									
30–39	169	5	(3.0)	1				-	-
40 and above	168	5	(3.0)	1.0	(0.3-3.4)	0.99			
**Gender**									
Men	212	08	(3.8)	2.4	(0.5-10.9)	0.3		-	-
Women	125	02	(1.6)	1					
**Residence**									
Urban	151	05	(3.3)	1.2	(0.4-4.2)	0.7		-	-
Rural	186	05	(2.7)	**1**					

aRR, adjusted relative risk; CI, confidence interval

***Qualitative***. Opportunistic screening was acknowledged by HCPs and persons screened for DM and HTN as a useful strategy for early detection. We have summarized the potential enablers and barriers for implementation of opportunistic screening for DM and HTN under two broad organizing themes, HCP-related (health care staff of the PHCs and District Programme Coordinator, NPCDCS) and patient-related (persons screened for DM and HTN from March to May 2019 in the two PHCs)( Figure 3 and Figure 4).

### HCP-related enablers

***Knowledge of screening***. The HCPs demonstrated satisfactory knowledge of the process and relevance of screening and acknowledged its role in early detection.

*“Now they (adult population) are coming early. Early screening is better because the disease onset is early and if undetected, could lead to complications.” (HCP, 34 years, female)*

***Willingness to deliver services***. HCPs expressed willingness to implement opportunistic screening in their settings, despite facing challenges like staff shortages.

*“We have trained all staff including attendants on Glucometer usage. Therefore, the screening is going on smoothly despite staff shortage.” (HCP, 55 years, male)*

Further, the HCPs believed that it is imperative to screen more often. One HCP stated that it is prudent to screen persons for DM and HTN at least once in six months.

*“We need to change to once in six months screening as once a year is inadequate. Over a period of time, the risk may increase.” (HCP, 44 years, female)*

***Strength of PBS***. PBS is being implemented in the community by the ANMs and ASHAs, through which many persons are being screened.

*“I strongly feel that PBS is the strength of NPCDCS at least in our district.” (HCP, 38 years, female).*

***Changes for better documentation***. Many HCPs welcomed the changes made in the OPD and NCD registers and were of the view that this improved the documentation of opportunistic screening.

*“It is absolutely fine. We get to know the eligible patients who really require screening.” (HCP, 29 years, female)*

Most of them did not experience any problems in recording the details mentioned in the registers.

*“No issues with the new documentation system. I could maintain both the registers properly.” (HCP, 29 years, female)*

### Patient-related enablers

***Awareness of screening***. Persons who underwent screening understood its role in early detection, facilitating prompt treatment and preventing complications.

*“The earlier we get diagnosed, the sooner we are treated. We must get screened before [the blood sugars and pressure]become high.” (patient, 52 years, male)*

***Readiness to undergo screening***. Most of the participants, expressed readiness to undergo screening, despite their values falling within normal limits. Many were willing to come for confirmatory tests if required. One participant indicated that she has followed dietary advice as a prevention for DM and HTN.

*“I get tested frequently even though I do not have disease. I have controlled my food habits just to be careful.” (patient, 60 years, female)*

***Satisfaction with health care***. Many persons were satisfied with the screening services. Two persons mentioned that they had not faced problems while undergoing screening at the PHC and were happy with the attitude of the HCPs.

*“No problems here. All staff are good.” (patient, 61 years, male)*
*“I haven’t faced problems. They check blood pressure and sugar properly.” (patient, 44 years, female)*

In spite of acceptance of this initiative by both HCPs and persons undergoing screening, several implementation barriers were noted.

### HCP-related barriers

***Staff challenges***. Many HCPs acknowledged increase in workload and inadequate staff as significant implementation challenges. Vacant posts, high proportion of persons with DM and HTN and the pressure of other health programmes were perceived to be the challenges.

*“Too many programmes and many patients. We are expected to check weight and height as well. Where do we get the time for all that?” (HCP, 29 years, female)*
*“Inadequate staff is a huge concern. Many PHCs do not have adequate staff nurses and laboratory technicians.” (HCP, 38 years, female)*

Lack of adequate human resources is the main issue, as stated by one HCP. To address this, task shifting is being practised with multiple personnel involved in documentation and screening.

*“We do not have enough staff. The data entry operator was on maternity leave for a long time. Data entry is now done by staff nurse as the data entry operator is overburdened. When she is not there, the laboratory technician or pharmacist contribute.” (HCP, 44 years, female)*

***Logistical issues***. Some HCPs expressed concerns over shortage of strips (Glucometer) and medicines once in a while. One HCP felt that facilities to transport patients/blood samples to a higher centre should be available, in case of non-availability of diagnostics at PHC.

*“Another issue is the shortage of NCD drugs. I end up prescribing for fifteen days instead of a month, which is not ideal.” (HCP, 44 years, female)*

Timely allocation and release of budget is another major barrier. Administrative delay was a contributing factor to the delay in release of funds and supply of equipment.

*“If we don’t have money, the implementation becomes difficult. Last year, the budget was approved on time but the money came only in December.” (HCP, 38 years, female)*
*“Things do not come on time and government procedures are lengthy.” (HCP, 55 years, male)*

***Documentation and reporting challenges***. High patient load and lengthy reporting format were the documentation-related challenges, as stated by a HCP. Another HCP suggested recruitment of dedicated staff for documentation and reporting.

*“The reporting format is complicated and consumes a lot of time.” (HCP, 35 years, female)*
*“The registers given by the programme are lengthy. It includes not just diabetes and hypertension but other NCDs like breast, cervical and oral cancers. All our staff are busy with other health programmes.” (HCP, 32 years, female)*

Delay in submission of reports was another issue that was highlighted by a participant.

*“We don’t get the reports on time. Reports have to reach by the 5 ^th^ of every month. But there is always a delay.” (HCP, 38 years, female)*

***Inadequate training***. Training conducted at the District NCD Cell focused more on treatment and indent of logistics while issues like screening were neglected. Need to organize comprehensive training programmes on screening and documentation was noticed.

*“I was not trained on how to conduct screening. The training focused on treatment and not on documentation and screening.” (HCP, 32 years, female)*

***Frequent screening demand***. Few HCPs opined that patients demand tests frequently. Thus, it is difficult to restrict screening to once a year.

*“If we make it (screening) once a year, many go and complain. If we do not agree to the patients’ demands, they complain to the corporator (elected public representative). I wonder if this would work.” (HCP, 29 years, female)*

***Low uptake among women***. HCPs stated that few women expressed difficulties in undergoing FBS the next morning.

*“Women give excuses and don’t turn up the next morning for FBS, despite we counselling them.” (HCP, 35 years, female)*
*“Women mention they have household work and refuse tests. I also feel that they are more anxious.” (HCP, 55 years, male)*

### Patient-related barriers

***Reluctance for FBS***. Many persons with high random blood glucose did not undergo FBS. One person felt that since glucometer testing is done, FBS on the next morning may be redundant. Another person stated preoccupation with work and late opening time of the centre as reasons for refusal.

*“I get it (sugars) checked in Glucometer, so what is the need of fasting sugars?” (patient, 50 years, female)*
*“PHC opens only at 9 am. I have to report for my work at that time.” (patient, 52 years, male)*

***Waiting time and inconvenience***. Few persons expressed their unhappiness about the waiting period for reports and consulting the MO.

*“By the time I finish my household work and come to the centre, the senior doctor would have left. Then, I have to wait for the next doctor in the evening (evening clinic).” (patient, 39 years, female)*

Two of the participants were apprehensive about the health care staff drawing the blood repeatedly, which caused them inconvenience.

*“Sometimes, they check our sugars despite getting it done recently. Why unnecessary take blood and subject us to more stress? ” (patient, 50 years, male)*
*“They prick thrice to collect blood. I fast overnight. It is difficult to withstand.” (patient, 46 years, female)*

***Challenges faced by women***. A least two women mentioned that it is difficult for them to make repeat visits to the PHC for testing, especially in the morning.

*“Here, it opens very late, at around 9 am. It is difficult for me, as I need to drop my children to school.” (patient, 50 years, female)*
*“I have to go for work at a factory after the household work. So, how will I be able to come in the morning?” (patient, 37 years, female)*

***Shortage of medicines***. Persons diagnosed with DM/HTN and started on treatment expressed concern regarding shortage of medicines for DM and HTN in the PHC.

*“They prescribe medicines for just 10–15 days and ask us to come back. It is a disturbance for us.” (patient, 47 years, male)*
*“They say there are no medicines here. They do not give for more than a week.” (patient, 61 years, male)*

De-identified transcripts from interviews and FGDs are available as *Underlying data* ^26^.

## Discussion

To our knowledge, this is one of the first mixed methods studies from India assessing the implementation of opportunistic screening for DM and HTN under NPCDCS in primary care settings. We made certain amendments in the OPD register to capture the population eligible for screening and in the NCD register to determine the number screened, diagnosed and treated for DM and HTN. We found that 19% were eligible for DM screening, of which 78% underwent screening and 13% were eligible for HTN screening, among whom 97% were screened. Willingness for screening both on the part of HCPs and persons seeking health care was a key facilitator. Several barriers like staff, logistics, documentation and waiting time were noted. The key findings are discussed below.

First, we found that a substantially low proportion were eligible for opportunistic screening (19.0% for DM and 13.0% for HTN). More than half were screened for DM and HTN in the last year. This is probably due to the PBS conducted in the rural community, an ongoing activity under carried out by ANMs/ASHAs who approach persons aged ≥30 years in the community through home visits or outreach camps. Community-based assessment checklists (CBAC) are filled out and those with high risk are referred to the SC for screening. If found positive, they are referred to the PHC for further investigations and treatment ^27^. Further, in urban areas of Mangaluru, special outreach camps with a focus on screening for DM and HTN are carried out once a month, which could have contributed to our finding of low proportion of eligible population.

Second, nearly 22% of the population screened were diagnosed with DM and 19% were diagnosed with HTN, which is much higher than the National Family Health Survey-4 (NFHS-4) data for DK district, in which ≈7.0% had high blood sugar and ≈12.0% had hypertension ^28^. This could be ascribed to the fact that our study was a facility-based assessment while NFHS-4 was a community-based survey. Similarly, a community-based survey in coastal Karnataka reported the prevalence of DM to be 16%, lower than the yield in our study. (19) A study conducted in a semi-urban population of Mangaluru reported a prevalence of 41% hypertension, which was much higher than our finding ^29^. Despite these variations, the high burden of DM and HTN is a matter of concern which requires both population and individual level interventions.

Third, women were more likely ‘not to be screened’ for DM when compared to men. This finding of our study could be attributed to the fact that women may be preoccupied with household work. This was substantiated in the qualitative component, where women listed reasons like domestic work and looking after children for not undergoing FBS. It could also be speculated that women are more likely to prioritize their family and may tend to neglect their own health. A qualitative study which assessed the barriers for screening of DM among Iranian women found that many women perceived screening for DM as difficult and also expressed reluctance to undergo blood sugar testing ^30^.

Fourth, we found that both the PHCs were staffed by HCPs who displayed a positive attitude towards delivery of NCD screening services. We also found that many persons seeking health care expressed readiness to undergo screening. Willingness is an important predictor for the success of screening for DM and HTN, as reported by previous studies ^31, 32^. The key reason for this finding could be the good rapport that the HCPs shared with the community.

Fifth, most of the HCPs were satisfied with the amendments made in both the OPD and NCD registers and believed that this made their job easier in terms of determining the eligible population. One drawback of the registers prescribed by the programme is that the eligible population could not be identified. The HCPs felt that the NCD registers prescribed by the programme include too many variables. We have tried to address this through modifications in the recording registers.

Sixth, few HCPs recommended half-yearly screening for persons without DM and HTN. This would lead to unnecessary screening and wasted resources There is a need to sensitize HCPs on restricting to once a year screening for judicious use of resources. The NPCDCS training manuals also advocate screening once a year for DM and HTN among the general population ^21, 27^. This needs to be emphasized in future training programmes conducted under the NPCDCS.

Seventh, staff challenges, logistical issues and documentation issues were the major barriers, as perceived by HCPs. The health care staff seem to be overburdened with many programmes. This is likely to affect their productivity and in turn hamper the implementation of opportunistic screening. Further, timely submission of reports to the district NCD cell becomes difficult.

Eighth, despite being aware of the relevance, many eligible persons failed to get themselves screened. Moreover, many who screened positive for DM did not undergo FBS. This was mainly due to preoccupation with work in the morning hours. Fear and uncertainty surrounding test results may have further contributed to this attrition. It is imperative to sensitize persons seeking care about the importance of FBS as a diagnostic test.

Increased waiting time was another challenge. It was also noted that laboratory technicians get deputed to other PHCs on certain days to address the issue of staff shortage. This may affect timely reporting of tests like FBS, which in turn results in a missed opportunity to initiate prompt treatment of DM.

### Strengths

This is the first study providing information on persons eligible for opportunistic screening in a primary care setting. Our study was conducted under programmatic conditions and the findings reflect the ground realities. We have used a sequential mixed-methods design, which helped in a comprehensive assessment of the enablers and barriers for implementation to guide further refinement of the programme. This will guide the programme managers to take corrective measures.

Most of the studies on this topic are focussed on population-based screening approaches and do not highlight facility-based implementation challenges. Since the investigators were not a part of the programme implementation team, this ensured objectivity in analysis and interpretation. Further, we included all persons aged ≥30 years seeking health care from the two PHCs, thereby ensuring internal validity. We adhered to the Strengthening the Reporting of Observational Studies in Epidemiology (STROBE) and COREQ guidelines for reporting quantitative and qualitative components, respectively ^25, 33^.

### Limitations

The findings of our study need to be interpreted cautiously as it was conducted in two PHCs.The findings cannot be extrapolated to other settings or geographical areas. There were some gaps in accurately recording the information about having underwent opportunistic screening in the last one year. There could be an element of recall bias as this was a self-reported variable.

### Program implications

First, urgent attention should be given to address staff challenges which includes filling of vacant posts and hands-on training for documentation. Second, we need to capitalize on the health seeking behaviour of persons seeking health care by timely delivery of services. Third, we need to nurture positive attitudes in HCPs by supportive supervision, training, regular supply of medicines and provision of incentives. Fourth, some eligible beneficiaries werenot screened. This needs to be addressed by digital solutions like line listing of the eligible population. Fifth, the modifications that we made in the registers helped in identifying the eligible population. However, this needs cautious interpretation and may require further studies before being implemented across all PHCs.

## Conclusion

Our study found a low proportion eligible for DM and HTN screening. Among those screened, a high number had DM and HTN. We made modifications in the documentation of screening which were well-received by the HCPs. We observed several enablers and barriers to implementation of opportunistic screening. The NPCDCS must address the barriers if it has to strengthen opportunistic screening in primary care settings.

## Data availability

### Underlying data

Figshare: Opportunistic screening for diabetes mellitus and hypertension in primary care settings of Karnataka, India: few steps forward but still some way to go- Raw Data.

http://doi.org/10.6084/m9.figshare.12052950 ^26^.

This project contains the following underlying data:

- Opportunistic screening DM HTN_OPD spreadsheet. (Data of 2697 persons who availed primary health care extracted from the OPD registers of two PHCs)
- Opportunistic screening DM HTN_NCD spreadsheet. (Data of 529 persons who were eligible for diabetes mellitus/hypertension screening extracted from the NCD registers of two PHCs)
- Opportunistic screening DM HTN_Data Documentation Sheet. (Data Documentation sheet used to code the variables)
- Key Informant Interview-Programme Coordinator. (Transcript of the Key Informant Interview conducted on the Programme Coordinator of the district)
- Key Informant Interview-Medical Officer 1. (Transcript of the Key Informant Interview conducted on Medical Officer of one of the two selected PHCs)
- Key Informant Interview-Medical Officer 2. (Transcript of the Key Informant Interview conducted on Medical Officer of one of the two selected PHCs)
- Key Informant Interview-Medical Officer 3. (Transcript of the Key Informant Interview conducted on Medical Officer involved in the non-communicable disease programme)
- Key Informant Interview- Staff Nurse 1 (Transcript of the Key Informant Interview conducted on Staff Nurse of one of the two selected PHCs)
- Key Informant Interview- Staff Nurse 2 (Transcript of the Key Informant Interview conducted on Staff Nurse of one of the two selected PHCs)
- Key Informant Interview- Staff Nurse 3 (Transcript of the Key Informant Interview conducted on Staff Nurse of one of the two selected PHCs)
- Key Informant Interview- Staff Nurse 4 (Transcript of the Key Informant Interview conducted on Staff Nurse of one of the two selected PHCs)
- Key Informant Interview- Laboratory Technician 1 (Transcript of the Key Informant Interview conducted on Laboratory Technician of one of the two selected PHCs)
- Key Informant Interview- Laboratory Technician 2 (Transcript of the Key Informant Interview conducted on Laboratory Technician of one of the two selected PHCs)
- Focused Group Interview-Patients 1 (Transcript of the Focused Group Discussion conducted on persons screened for diabetes mellitus or hypertension in one of the two selected PHCs)
- Focused Group Interview-Patients 2 (Transcript of the Focused Group Discussion conducted on persons screened for diabetes mellitus or hypertension in one of the two selected PHCs)
- Focused Group Interview-Patients 3 (Transcript of the Focused Group Discussion conducted on persons screened for diabetes mellitus or hypertension in one of the two selected PHCs)
- Focused Group Interview-Patients 4 (Transcript of the Focused Group Discussion conducted on persons screened for diabetes mellitus or hypertension in one of the two selected PHCs)
- Focused Group Interview-Patients 5 (Transcript of the Focused Group Discussion conducted on persons screened for diabetes mellitus or hypertension in one of the two selected PHCs)

Data are available under the terms of the Creative Commons Attribution 4.0 International license (CC-BY 4.0).

### Extended data

Figshare: Opportunistic screening for diabetes mellitus and hypertension in primary care settings of Karnataka, India : few steps forward but still some way to go- Extended Data

http://doi.org/10.6084/m9.figshare.12053055 ^20^.

This project contains the following underlying data:

- Study Proforma (Data collection proforma used in the study)
- Key Informant Interview Checklist (Checklist used for the Key Informant Interviews)
- Focused Group Discussion Guide (Guide used for the Focused Group Discussions)

Data are available under the terms of the Creative Commons Attribution 4.0 International license (CC-BY 4.0).
