# Supplementary material for: Opportunistic screening for diabetes mellitus and hypertension in primary care settings in Karnataka, India: a few steps forward but still some way to go
Source: F1000Res. 2020 May 6;9:335. [Version 1] doi: 10.12688/f1000research.22825.1 (PMC7676396; doi:10.12688/f1000research.22825.1)
Supplement: Peer review history

# Peer review: Reviewer response for version 1

**DOI:** 10.5256/f1000research.25200.r70384

**Published:** 2020-10-08

**Authors:** Mongal Singh Gurung

**Affiliations:** 1 Health Research and Epidemiology Unit, Ministry of Health, Thimphu, Bhutan

This is a very important operational research that provides insights for enhancing opportunistic screening and management of DM and HTN in Indian settings and other developing countries. Overall, the article is well written and merits indexing.

 I have the following suggestions/clarification:

1. The first sentence for results under the abstract could be split into two sentences for clarity and to segregate the findings by the study objectives. The second of the split sentences could present figures for cascades of screened, diagnosed, and initiated on treatment. The same comment applies to the HTN in the second sentence.
2. According to the information provided under the study population section, it looks like all healthcare providers were interviewed but under Phase 2: Qualitative data collection section it says “… until information saturation was attained” as if recruitment of participants for the qualitative part were stopped when information saturation was attained." This may need clarification.
3. How many participants did not consent for audio recording? I suggest providing the exact number of participants who did not consent to the audio recording.
4. Providing justification(s) for the choice of the KII and FGDs methods used may be useful for readers.
5. How many FGDs were conducted?
6. In Figure 2 the box “High Blood Pressure (SBP ≥140 and /or DBP ≥90) = 63/327(19.3%)” could be rephrased as “High Blood Pressure **on screening** (SBP ≥140 and /or DBP ≥90)=63/327(19.3%)” for clarity. Maybe the box “diagnosed as HTN =8/63(12.7%)” is “ **Not **diagnosed as HTN =8/63(12.7%)”.
7. aRRs were calculated only for DM and not for HTN. Further, I think we adjust or control for other variables in the multiple variable models instead of “eliminate the confounders”. Only three variables (viz. age group, gender, and residence) were included in the models.
8. There were only 10 eligible individuals who were not screened for hypertension and only 2 women were not screened. This analysis may best be done away with. If Table 4 is retained then the last three columns of Table 4, which are empty and not relevant, could be removed.
9. Table 2 shows information about the study participants, not all “persons aged ≥30 years seeking health care”. Therefore, the table title may need to be rephrased for clarity.
10. Gender and sex are very related words but they have different meanings. Usually, the administrative data of health is on sex rather than gender. Therefore, I suggest a review of its usage in the manuscript.
11. Not including those who were not screened, in the qualitative interviews, to understand the barriers seems to be one of the limitations of this study.  
12. Few typos:
  1. The was done to estimate the number of persons eligible for screening and to assess the feasibility of this strategy in such settings. 
  2. A total of 6–8 participants were included in each FGD. FGDs.
  3. Of the 2697, 812 (30.2%), were aged 30–39 years with a mean age of 47.7 years (SD:12.3 years).

Is the work clearly and accurately presented and does it cite the current literature?

Yes

If applicable, is the statistical analysis and its interpretation appropriate?

Partly

Are all the source data underlying the results available to ensure full reproducibility?

Yes

Is the study design appropriate and is the work technically sound?

Yes

Are the conclusions drawn adequately supported by the results?

Yes

Are sufficient details of methods and analysis provided to allow replication by others?

Partly

Reviewer Expertise:

Noncommunicable Diseases (NCD) and Medical Statistics

I confirm that I have read this submission and believe that I have an appropriate level of expertise to confirm that it is of an acceptable scientific standard, however I have significant reservations, as outlined above.

# Peer review: Reviewer response for version 1

**DOI:** 10.5256/f1000research.25200.r64187

**Published:** 2020-06-19

**Authors:** Subita P. Patil

**Affiliations:** 1 Department of Preventative Oncology, Tata Memorial Centre, HBNI, Mumbai, Maharashtra, India

- The article is written very well.
- IMRAD format explained appropriately.
- The design of the study is a good mix of qualitative & quantitative methods in current settings.
- The conclusion, the strengths of the study are well narrated.
- The outcome of the study is service-oriented for the benefit of the community.
- The study can be accepted as it is.

Is the work clearly and accurately presented and does it cite the current literature?

Yes

If applicable, is the statistical analysis and its interpretation appropriate?

Yes

Are all the source data underlying the results available to ensure full reproducibility?

Yes

Is the study design appropriate and is the work technically sound?

Yes

Are the conclusions drawn adequately supported by the results?

Yes

Are sufficient details of methods and analysis provided to allow replication by others?

Yes

Reviewer Expertise:

Epidemiology, public health,  preventive oncology, medical education

I confirm that I have read this submission and believe that I have an appropriate level of expertise to confirm that it is of an acceptable scientific standard.

# Peer review: Reviewer response for version 1

**DOI:** 10.5256/f1000research.25200.r63174

**Published:** 2020-05-14

**Authors:** Anindo Majumdar

**Affiliations:** 1 Department of Community and Family Medicine, All India Institute of Medical Sciences (AIIMS), Bhopal, Madhya Pradesh, India

**Summary of the report:**

 The authors have conducted a timely and much needed research which was lacking even after about a decade of implementation of the NPCDCS. The use of mixed methods was required and quite rightly used for understanding such a complex process. 

 The main issue with the current manuscript looks to be a confusion between two research questions:

1. What is the number and proportion eligible for screening of DM and HTN and subsequently, among those eligible, how many were screened, diagnosed and managed for the disease under routine programmatic field conditions?
2. What effect does improving the existing documentation system have on the above mentioned variable and can the new system make documentation and reporting better?

The issue is, both are different research questions and trying to answer both of them in a single study will require better clarity in presentation and accepting the limitations of the study which will increase in number as a result of doing so. Nevertheless, since the issue is very important, with the incorporation of the suggested changes, the manuscript has the potential to be a very good one. 

**Full report:**

 Some specific issues are highlighted below:

**(A) Abstract **

How is it a cohort design? There is no clear exposure. If we take age, gender and residence as exposure and outcome as screened for which analysis has been done, all these variables were collected at the same time for each participant with respect to the participants time. The quantitative part of this study has a cross-sectional design and should be mentioned as cross-sectional.
a) In results give denominator for 401.
  b) For the sentence - 'The documentation changes helped in identifying the eligible population' - this needs to be reworded to bring more clarity. 
  c) Is willingness not a result of other factors such as recognition of relevance of screening rather being an independent enabler in itself?

**(B) Main manuscript:**

1. Under Study Population subheading - How was the sample size for qualitative study reached at? What was the sampling technique used? These need to be mentioned.
2. Under subheading - Data variables, sources of data and data collection - While improving the system by developing better documentation systems has helped the researchers to get to the number screened, diagnosed and put on treatment, the qualitative part would not correctly reflect on the real field level functioning of the health centres under programmatic settings. For instance, some participants of in-depth interviews might liked to have mentioned the original documentation system (before improvement) as one of the main barriers. Since introducing the change in the recording system and seeing its outcome was not the objective, this should be mentioned as one of the limitations of the qualitative part. 
3. Under data collection subheading - Were interns posted also taken as HCWs and interviewed?
4. Under Phase 2: Qualitative data collection subheading:
  a) Since the PI himself interviewed the health workers of health centres, which he supports administratively, there is a definite element of bias while the HCWs would have given their views. Also, during the initial improvement of the documentation systems, the same stakeholders would have participated. It is quite possible they got motivated during this phase which would have had an impact on their responses. Also, they understood the study objectives during this phase, which would have also had an impact on their responses. This should be highlighted as a limitation. 
  b) It is not clear how many KIIs and FGDs were conducted initially. This should be mentioned.
5. Under Statistical and data analysis subheading - Analysis should be as per cross-sectional study design and not cohort.
6. In Figure 2 - some statements are incomplete/incorrect:
  a) Care at PHC OPD - Initial part of the statement is missing.
  b) Diagnosed as HTN-8/63 - I think this should be 'not diagnosed as HTN'.
7. Under results section:
  Quantitative - Testing the association of socio-demographic characteristics with not being screened. Was this also a study objective?
  Qualitative:
  a) “We have trained all staff including attendants on Glucometer usage. Therefore, the screening is going on smoothly despite staff shortage.”(HCP, 55 years, male) - Was this training given before the study or after they came to know about the study as a result of initial documentation improvement discussions? Since this would influence the results. Clarity is needed on this.
  b) Strength of PBS - It needs to be explained how this links to the study objectives that are related to opportunistic screening and not PBS.
  c) Patient-related enablers - Awareness of screening - Only persons who underwent screening have been interviewed. Since quantitative analysis was done to understand the factors associated with those 'not screened', it was equally important to know their perspective in qualitative interviews. This should be mentioned as a limitation.
  d) Documentation and reporting challenges - It needs to be explained more as the HCPs have also found the new reporting system as an enabler, although it would have made the new documentation/reporting format a bit more lengthy. This discrepancy needs to be explained well in the discussion part. 
  e) Patient related barriers - Waiting time and inconvenience barrier and the reluctance for FBS barrier is contradictory to patient related enablers which has listed greater readiness to undergo screening and awareness and satisfaction with services. This needs to be explained in discussion section. 
  f) In the limitations section - Also, asking the patient if the patient has already got DM/HTN is subject to wrong recoding of information if it was only based on verbal information from patients. Unless documentary evidence was sought to confirm, this should also be mentioned as a limitation.

Is the work clearly and accurately presented and does it cite the current literature?

Partly

If applicable, is the statistical analysis and its interpretation appropriate?

Partly

Are all the source data underlying the results available to ensure full reproducibility?

Yes

Is the study design appropriate and is the work technically sound?

Partly

Are the conclusions drawn adequately supported by the results?

Yes

Are sufficient details of methods and analysis provided to allow replication by others?

Partly

Reviewer Expertise:

Noncommunicable disease

I confirm that I have read this submission and believe that I have an appropriate level of expertise to confirm that it is of an acceptable scientific standard, however I have significant reservations, as outlined above.
